# Barnum’s Coffer-Dam

**Published:** 1874-07

**Authors:** 


### BARNUM'S COFFER-DAM.

Dr. S. C. Barnum being the discover of the application of sheet rubber, for keeping the natural teeth dry when operating upon them, we would suggest, that hereafter the article be called by all the profession the Barnum's Rubber-Dam, as this seems to be the proper name, at once signifying what the material is, and also its use. As has been often stated, the Dr. has not been at all mercenary, making no demands whatever upon us, in this he has taken the highest standard of regard for his brethren and love for his profession; thus has he seemed to care more for doing good, and having the best wishes of his profession, rather than to have money. So out of re-pect for him, and that his memory be perpetuated, showing a worthy example for others, let us say Barnum's Rubber-dam. From what we have read and heard, the Dr. will esteem this recognition much more than all the money and medals he has already received. When coming generations take our places, according to the nature of things, should it be asked who was the discover of Rubber-dam. The reply would be, well I do not just remember, but it is a good thing. Now this can all be avoided by adopting the above suggestion, and we at the same time acknowledge our high appreciation of the Dr. and his discovery. A CONTRIBUTOR.

